# Applications of Piezoelectric Materials in Structural Health Monitoring and Repair: Selected Research Examples

**DOI:** 10.3390/ma3125169

**Published:** 2010-12-06

**Authors:** Wen Hui Duan, Quan Wang, Ser Tong Quek

**Affiliations:** 1 Department of Civil Engineering, Monash University, Clayton, Victoria, 3800, Australia; E-Mail: wenhui.duan@monash.edu; 2 Department of Mechanical and Manufacturing Engineering, University of Manitoba, Winnipeg, Manitoba, R3T 5V6, Canada; 3 Department of Civil Engineering, National University of Singapore, 119596, Singapore; E-Mail: cveqst@nus.edu.sg

**Keywords:** structural health monitoring, structural repair, piezoelectric sensors and actuators, wave propagation, interdigital transducer, wavelet transform

## Abstract

The paper reviews the recent applications of piezoelectric materials in structural health monitoring and repair conducted by the authors. First, commonly used piezoelectric materials in structural health monitoring and structure repair are introduced. The analysis of plain piezoelectric sensors and actuators and interdigital transducer and their applications in beam, plate and pipe structures for damage detection are reviewed in detail. Second, an overview is presented on the recent advances in the applications of piezoelectric materials in structural repair. In addition, the basic principle and the current development of the technique are examined.

## 1. Introduction

The assessment of the performance of structures in terms of ultimate and serviceability limit state, durability and catastrophic failure has always been an important issue. Early detection of anomaly such as defects or damages in a structure is necessary for optimal decisions with regards to its rehabilitation, strengthening, and/or reconstruction. This has led to two interrelated research topics, *i.e.,* structural health monitoring and structural repair.

Many practical and robust non-destructive evaluation (NDE) techniques for structural health monitoring at the element level have been developed based on wave propagation signals [1,2,3]. These include monitoring changes in modal parameters (e.g., natural frequencies, modal damping, mode shapes), ultrasonic scanning using propagating waves in structures (e.g., impulse-echo technique), and the recent imaging techniques using advanced equipments such as infra-red (thermo-graphic inspection) or laser (radiographic inspection) scanning. Nonetheless, damage detection at both structural and element level still poses considerable challenge [4]. Ultrasonic inspection technique using propagating wave signals can be considered as one of the most commonly used techniques and its application has been promoted rapidly over the past two decades due to the corresponding advancement in electronic equipment, which makes the technique practical, cheaper and readily available. Recently, piezoelectric actuators and sensors [5,6,7,8,9,10,11,12,13,14,15,16,17,18,19,20,21,22,23,24,25,26] have been widely used in damage detection of various structures including beams, plates and pipes due to their unique sensing and actuating properties. Waves in solids are usually initiated by an impact force on the surface of the media, which could be modeled as a pulse. The convenience of this excitation method is often negated by the wide frequency band and the uncontrollable amplitude of the generated signal giving inconsistent results. Alternatively, the advantage of piezoelectricity can be made useful in exciting elastic waves using electrical signal input. Both requirements of convenience and precision in wave excitations are satisfied using this mode of actuation. In addition, piezoelectric sensor is much more consistent and reliable than conventional strain gauge to determine damage location, since piezoelectric sensors are self-powered [5].

On the other hand, structural repair has been a topic of concern. Unattended structural damage can grow at an alarming rate due to the singularity in stresses and strains near the damage region. The damage can lead to increased vibration level [27,28], reduction in load carrying capacity [29,30], deterioration in performance of the component [31,32], and even catastrophic failure. Under most conditions, the service life of damaged components may be prolonged through cost-efficient repair instead of an immediate replacement. Therefore, effective repair of a structural damage is an important and practical topic. Conventional repair methods usually involve welding, riveting or mounting additional patches to the parent structure without removing the damaged portion, and their limitations have been reviewed [33]. The review highlighted the need for more effective repair methods using smart materials [34,35,36] to be developed, since the strains and stresses in areas surrounding the damaged domain may cause additional damage, which results in severe reduction in the service life of the entire structure.

The content of the paper is arranged as follows: in Section 2, the commonly used piezoelectric materials and their application are introduced. The rest of the paper focuses on the recent progresses and the applications of piezoelectric materials in structural health monitoring and repair conducted by the authors and their collaborators. The analysis of plain piezoelectric sensors and actuators and interdigital transducer and their applications in beam, plate and pipe structures for damage detection are presented in Section 3 and Section 4, respectively. “Plain” refers to a fully coated electrode on the piezoelectric sensors and actuators. The applications of piezoelectric materials in structural repair of delamination and notch crack in beam and plate structures are presented in Section 5.

## 2. Piezoelectric Materials and Applications

Piezoelectricity is a phenomenon in which mechanical energy is converted into electrical energy and vice versa. By definition, a material possessing piezoelectricity will generate an electrical charge when a mechanical pressure is applied to it. Likewise, the material will experience a geometric change when an electrical charge is applied to it. There are a few natural materials that exhibit piezoelectricity, of which piezoelectric ceramics (Lead Zirocondate Titanate, or in short, PZT), piezoelectric polymers (Polyvinylidene Fluoride, denoted as PVDF) and Piezoelectric Ceramic/Polymer Composites are frequently used piezoelectric actuators and sensors for structural health monitoring and structural repair. Gururaja [37,38] summarized the advantages and disadvantages of each type of the material: (1) Ceramics are less expensive and more easily fabricated than polymers. They have relatively high dielectric constants and good electromechanical coupling. Since they are stiff and brittle, monolithic ceramics cannot be coated onto curved surfaces, which limits the design flexibility in the transducer; (2) Piezoelectric polymers are very flexible but have limitations of low electromechanical coupling and low dielectric constant, and high cost of fabrication; (3) Piezoelectric ceramic/polymer composites have shown superior properties when compared to single phase materials. They have high coupling, low impedance, few spurious modes, and an intermediate dielectric constant.

The reciprocal energy transforming characteristics of piezoelectricity enables piezoelectric materials to function as sensors, actuators or transducers [12,17,21,22]. When they are used as sensors, the input mechanical signal is transformed into electrical signal that can be evaluated through electrical equipments. When they are applied as actuators, strains are produced to control the substrate behavior given an applied voltage. Finally, when they are used as transducers, the high frequency electrical input signal is transformed into mechanical wave. If the frequency of an electrical input is narrow band, the frequency band of the output mechanical wave remains narrow. The unique behavior of piezoelectric material makes it as a transducer to excite wave on the bonded substrate or as a sensor to detect the mechanical wave propagating in the substrate. Recently, Liang *et al.* [39] experimentally performed the coupled electrical-mechanical (E/M) analysis of adaptive systems driven by a surface-attached piezoelectric wafer, showing that the E/M admittance response accurately reflects the system dynamic response. Giurgiutiu *et al.* [40,41,42,43] attempted theoretical modeling on the piezoelectric sensor dynamics for various boundary conditions and its interaction with the host structure, indicating that E/M admittance or impedance frequency spectra can properly capture the changes in local dynamics due to incipient structural damage. As a result of the availability of piezoelectric materials with strong electromechanical coupling effect, new sensors and actuators involving piezoelectric elements have found wide applications and are in greater demand. Examples include piezoelectric ultrasonic motors [44,45], piezoelectric transducers for structural health monitoring [20,23,24], and vibration control or noise suppression using piezoelectric layer [46,47,48,49]. Subsequently, the structural coupling effects between the piezoelectric material and the host material become a topic of practical importance.

Piezoelectric materials, as an alternative to conventional materials in structural repair, open up new opportunities for improved repair techniques to overcome some of the limitations of conventional repair methods [34,35,36]. Piezoelectric materials allow performing active repairs, since their strength and their interaction with the repaired structure can be adjusted to compensate for environmental changes. Piezoelectric patches are also much lighter than conventional materials and create less concentrated stress on the damaged structure. Pioneering researches on repair of damaged steel structures by use of piezoelectric patches were conducted by Wang *et al.* [50,51,52,53,54,55,56,57,58]. The concept was also extended to the repair of reinforced concrete beams [59,60], damaged structures with a moving mass [61], and analyzed by analytical fracture mechanics [62] and boundary element methods [63].

In addition, there is significant work on so-called “smart patches” for structural health monitoring and structural repair. Smart patches have multiple functions, which include self-rehabilitation, self-structural health monitoring, and self-vibration damping. By taking the advantage of shape memory alloy (SMA) and PZT, the concept of intelligent reinforced concrete structure was proposed [64], in which SMA cables close the crack in the structure and embedded PZT patches are used to detect the onset and severity of cracks. Smart patches based on carbon fiber reinforced polymers with embedded PZT patches were investigated with the aims towards providing permanent and integral monitoring of structural and functional integrity as well as implementing as a structural health monitoring system using electrical impedance spectroscopy [65,66].

## 3 Analysis and Applications of Piezoelectric Sensors and Actuators

### 3.1. Plain Piezoelectric Sensors and Actuators

The pioneer work of analysis of piezoelectric sensors and actuators was done by Crawley and Luis [67]. Both analytical and experimental results of piezoelectric actuators as elements of intelligent structures, *i.e.,* structures with distributed actuators, sensors, and processing networks were provided. Analytical models for dynamic response were derived for patches of piezoelectric actuators bonded on an elastic substructure or embedded in a laminated composite. Their models were capable of predicting the response of the structural member at a given voltage applied to the actuators and providing guidance on optimal location of actuators. A scaling analysis was performed to evaluate the effectiveness of various piezoelectric materials in transmitting strain to the substructure. Lee and Moon [68] developed a set of piezo-polymer devices based on a piezoelectric polymer composite laminate theory. With different combinations of ply angles and electrode patterns, a piezopolymer/metal thin plate structure was built that exhibited both bending and torsion deformation under an electrical field. A set of torsion-beam sensor structures were also incorporated that could distinguish between bending and torsion vibration modes. They also performed experiments and achieved results in agreement with those from theoretical predictions. Besides being used for the control of structural behavior, piezoelectric materials are frequently used as transducers to excite acoustic waves. The study of wave propagation inside piezoelectric materials attracted much attention for the application of delay line. The time delay effect was achieved when a piezoelectric element is used to transform the electrical signal into the elastic wave propagating through it [69,70]. Sun and Cheng [71] studied acoustic wave propagating around a piezoelectric cylinder with thin metallic overlay and showed that the wave propagation characteristics changed with different metallic layers. Their investigation manifested the importance of evaluating the electro-mechanical effect in a layered structure. The piezoelectric coupled structures with regular shape, such as beams [7,8,72,73,74], circular plates [6,10,75,76], annular plates [77], cylinders [13,16,78,79], and shells [80] have been extensively investigated by authors and their co-workers.

**Figure 1:**
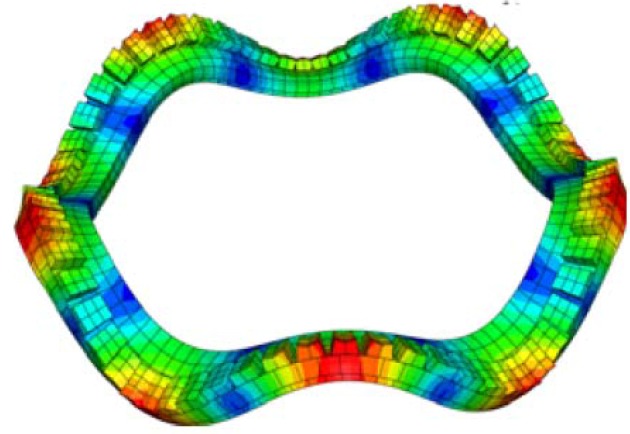
Snapshot on the transient response of a piezoelectric coupled ring with wave propagating (adopted from Figure 13d in [44]).

Researchers have also shown that closed-form solutions accounting for the electro-mechanical coupling of piezoelectric plates, laminates or patches are difficult to obtain especially for complicated boundary conditions. Thus an approximation technique, such as finite element (FE) method, is necessary. Variational methods and FE models for piezoelectric beams and plates have been reported by Tzou and Tseng [81], Ha *et al.* [82], Hwang and Park [83] as well as Lam *et al.* [84]. Recently, Duan *et al.* [44,45] conducted the analysis of wave propagation in a piezoelectric coupled structural ring via piezoelectric actuation using commercial software ABAQUS. 3D solid elements with 20 nodes (C3D20RE and C3D20R) were adopted for piezoelectric patch and structural ring to take into account the full piezoelectric coupling effect. The wave propagation of the piezoelectric coupled structural ring is shown in Figure 1.

### 3.2. Interdigital Transducer

Interdigital transducer (IDT) was first used to improve the performance of piezoelectric fiber composites [85,86] and subsequently to excite the Surface Wave devices in Radar communication equipment as filters and delay lines [87], and some consumer areas such as pagers, mobile phone, and sensors [88,89]. Its applications in separating, amplifying, and storing signals and in other signal processing applications in acousto-electronics were also considered important [90,91]. It should be noted that in these applications three important assumptions are normally employed to simplify the analytic models of IDT. Firstly, piezoelectric effects are assumed to be small enough that the electrical field can be calculated first and then applied to obtain the mechanical field. Secondly, infinite number of fingers is assumed to admit a fully periodic solution. Lastly, the piezoelectric substrate is assumed to be semi-infinite. Based on these three assumptions, the dispersive characteristics of wave propagation in various piezoelectric medium are derived. Curtis and Redwood [92] proposed a solution for dispersion characteristics of the shear-horizontal (SH) wave in a piezoelectric material of class 6 mm. The piezoelectric medium in their studies was surface bonded by a layer of metal. Feng and Li [93,94] and Kielczynski *et al.* [95] further provided analyses on the SH waves on piezoelectric ceramics with metal surface layer.

**Figure 2:**
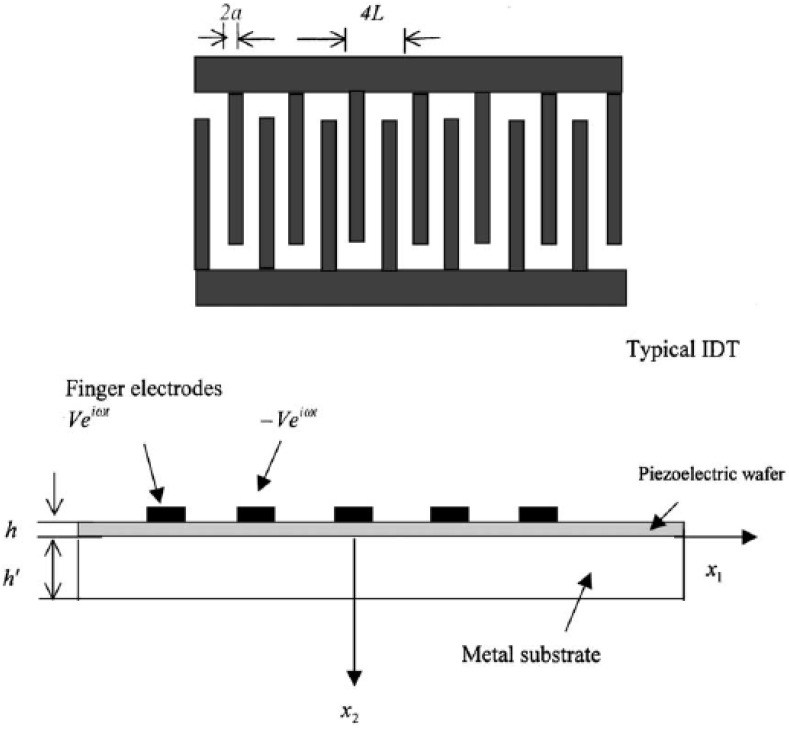
Piezoelectric coupled plate surface bonded by interdigital transducer (adopted from Figure 1 in [15]).

Nowadays, an important application field of IDT is in the health monitoring of structures. A practical IDT with finite length bonded by a metal plate is shown in Figure 2 [15]. IDT is a thin piezoelectric film surface bonded on either piezoelectric or unpiezoelectric substrate for the use of wave excitation or reception of structures. On the surface of the wafer, a pattern of electrodes is designed, which comprises two alternating sets of fingers connected to external electric power sources for the energy supply. Since IDT is bonded on a piezoelectric layer which is surface bonded on the metal substrate, the piezoelectric effects must be modeled in the dispersion characteristics of the piezoelectric coupled structure. Jin *et al.* [12] derived the lamb wave propagation in a metallic semi-infinite medium covered with piezoelectric layer. Wang *et al.* [11,96] studied the Love wave propagation in a semi-infinite metal medium with a piezoelectric layer mounted on the surface. The Bleustein-Gulyaev wave was observed for the first mode of the wave solution. Some other wave modes were also observed. The mode shapes of the electric potential and displacement were studied as well in their work. Wang and Varadan [14,15] derived the dispersion characteristics of the wave propagation of SH wave and the analytical solution for SH wave propagation excited by IDT in a piezoelectric coupled plate. Both infinitely long and finitely long IDT were modeled.

The key issue for application of IDT in structural health monitoring is how to design the size of the IDT, such as its wavelength and finger width, so that a wave signal with higher magnitude and less dispersive effect can be excited and sensed. The analysis of IDT is crucial in order to have optimum design. There are considerable researches on the subject of analysis of IDT, in which the combination of analytical and numerical methods were attempted [97]. The difficulties of the analyses lie in the full electromechanical coupling in the structure. Tseng [98], Coquin and Tierstan [99], and Joshin and White [100] analyzed this problem by solving an electrostatic problem, and substituted the distribution of the electric fields into the electromechanical coupled equation, hence obtaining the secondary electric fields and the distribution of displacement fields. In a monograph of Parton *et al.* [91], the analytical solutions for an IDT which generates Rayleigh surface waves in a hexagonal 6 mm piezoelectric medium were presented based on the same procedure. However, they could not fully model the piezoelectric effects in their models. Some recent progresses were contributed by the FE method [101,102], the boundary element method [21,103,104,105,106], finite difference method [107,108,109] and time-reversal of ultrasonic fields [110,111]. FE method has the advantage in modeling the complicated electrical and mechanical boundary conditions of IDT, and eliminates the need for lengthy analytical solution [112]. Al-Nassar *et al.*[113] combined the FE method to simulate the localized plate region containing weldment and Lamb mode expansion to represent the wave field in the remaining region of the plate. A similar procedure was used by Moulin *et al.* [114] to study the interaction of Lamb wave with defects and the excitation of Lamb wave by PZT patch, respectively. In both cases, only a region of the plate containing structural discontinuities is meshed with finite elements, while the known analytical wave propagation solution is applied on the remaining portion of the plate. The significantly reduced computation effort made the solution possible.

Although the FE method minimized the difficulties in modeling the complicated boundary conditions, it is a time-consuming technique in simulating wave propagation problem, in which the size of the element is of the order of the wavelength and the time step is usually as small as one microsecond. The FE solution for the near field is still computationally involved and time-consuming and convergence problem is still prevalent. A more practical analytical model was proposed by Jin *et al.* [17] to simulate IDT wave excitation in NDE, in which the aforementioned three assumptions are relaxed. Firstly, strong electro-mechanical coupling is considered; secondly, the number of fingers in the IDT considered is finite; and thirdly, the substrate is modeled as a plate instead of a semi-infinite medium. Perfect bonding between IDT and the host plate is assumed. Experiments were performed in an aluminum plate to obtain Lamb waves excited by IDT. The ratio of the amplitudes of different Lamb wave modes matches the acoustic fields obtained from the analytical method.

## 4. Structural Health Monitoring

Comprehensive methodologies [5,9,18,20,22,,23,26] for locating and determining the extent of linear crack in beams, plates and pipes have been developed based on the time-of-flight analysis of Lamb wave propagation via appropriate data analysis methods such as wavelet transform initiated by Wang *et al.* [115,116,117] and Hilbert-Huang transform (HHT) [118,119,120]. These methodologies and applications are reviewed hereinafter.

### 4.1. Beams

**Figure 3:**
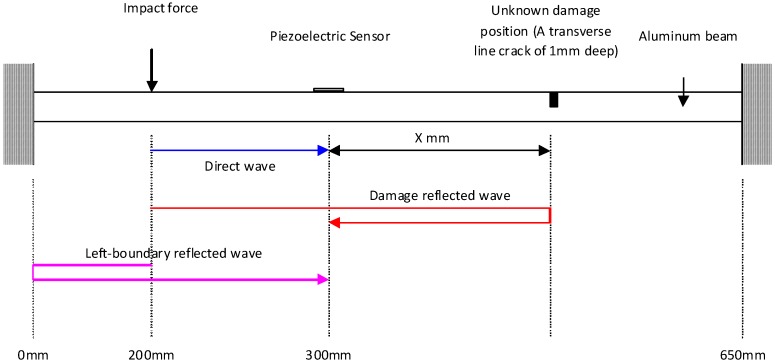
Experimental set-up to detect the transverse line crack in beam (adopted from Figure 6 in [18]).

**Figure 4:**
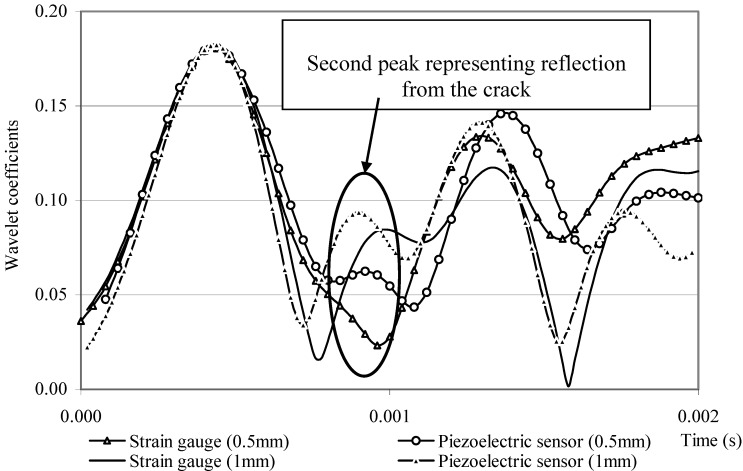
Wavelet coefficients at scale 12 of signals from piezoelectric sensor and strain gauge for 0.5 mm and 1 mm deep cracks (adopted from Figure 4 in [5]).

Quek *et al.* [5] presented the experimental data in locating a crack in an aluminum beam based on simple wave propagation considerations. The beam considered has a clear span of 650 mm, 32 mm width and 6 mm depth, with modulus of elasticity of 73.1 GPa and density of 2,790 kg/m^3^ as shown in Figure 3. As the choice of sensor is an important consideration, piezoelectric sensor and conventional strain gauge are attached at 300 mm (see Figure 3) for comparison purpose. The signals collected based on an impact force at 200 mm and 0.5 mm and 1.0 mm deep linear cracks at 450 mm are processed using wavelet transform. The plot of wavelet coefficients is shown in Figure 4. It can be seen that the second peak is clearly detected using the piezoelectric data whereas it is virtually non-existent using the strain gauge data. For a deeper crack of 1 mm, both types of sensor give distinct second peak. The results show that piezoelectric sensor is much more consistent and reliable than strain gauge for finding damage location and should be used wherever possible. Three sets of data with different boundary conditions are analyzed, *i.e.*, fixed-ended, simply-supported and cantilever conditions. Both piezoelectric sensors and conventional strain gauges capture the wave signal which contains timings of the direct, damage reflected and boundary reflected waves. By estimating these timings using signal processing technique, the damage location can be deduced. The results from experimental data indicated that wavelet transform of dynamic response data from piezoelectric sensor is suitable for detecting local damage in beams.

The same three sets of signals were also analyzed using HHT[18]. The results for the estimated damage position show that the HHT method is able to identify the damage position with reasonable accuracy, comparable to wavelet results. The advantage of HHT over wavelet technique in beam crack detection is that the HHT procedure is more direct whereas the wavelet transform is harder to apply.

**Figure 5:**
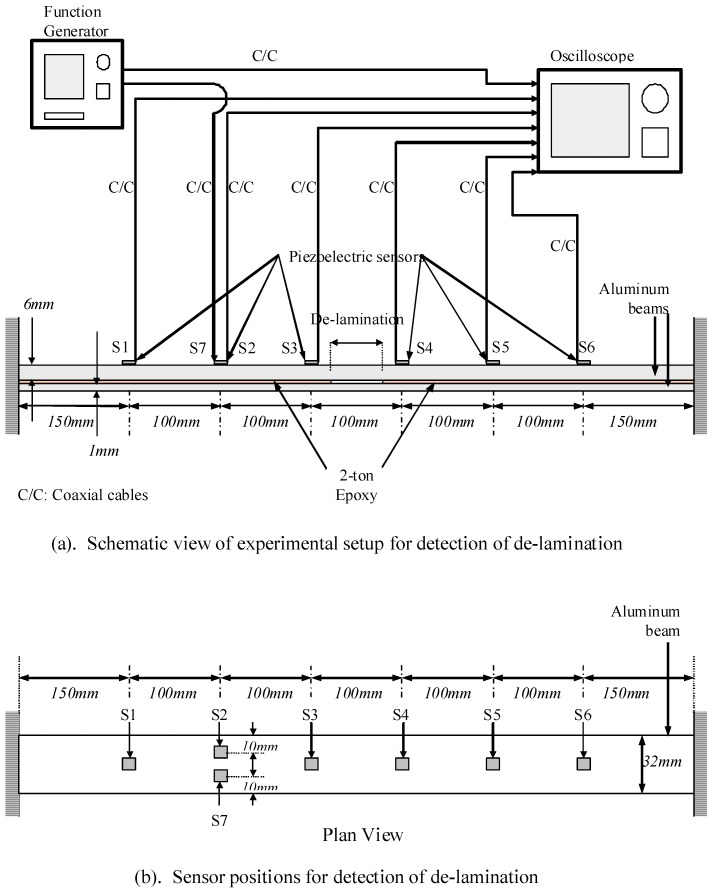
Experimental set-up to detect the delamination in beam (adopted from Figure 8 in [18]).

The piezoelectric sensors and actuator and HHT are also used in detecting the delamination in a beam as shown in Figure 5. The idea to detect the delamination is that when a wave propagates through a beam with delamination, the velocity through the intact section and the velocity through the delaminated section will be different. By sensing the wave at regular intervals along the beam, any section that is delaminated may be detected based on the arrival times of the impact wave. Two 32 mm wide aluminum strips with thickness of 6 mm and 1 mm are glued together throughout their length using epoxy with strength of 2-ton, leaving a 60 mm gap at the centre portion of the beam. The beam is set-up to have a span of 800 mm with six piezoelectric sensors S1–S6 and one actuator S7 attached along the beam, as shown in Figure 5. Co-axial cables are used to connect the sensors and actuators to the function generator and oscilloscope. The results show the wave velocity of 2,105 ms^−1^ across the healthy area and a velocity of 1,639 ms^−1^ through the delaminated area, indicating the effectiveness of piezoelectric sensors and HHT in the detection of delaminated beam.

**Figure 6:**
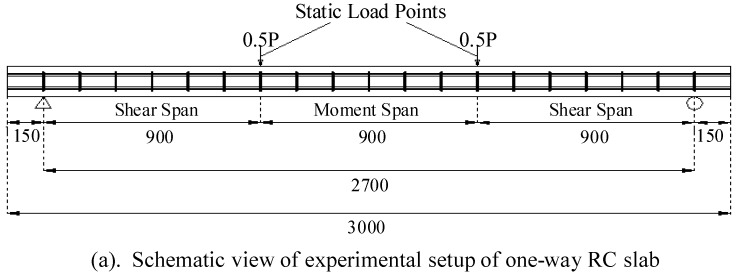

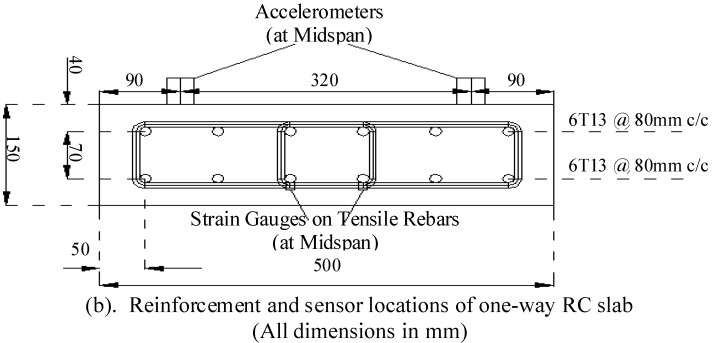
Experimental set-up to detect the reinforcement in one-way RC slab (adopted from Figure 12 in [18]).

Koh [121] experimentally studied the properties of a simply-supported one-way RC slab with a cross section of 500 mm by 15 mm and a span of 2,700 mm. The RC slab undergoes various degrees of damage when subjected to increasing magnitude of third point loads, namely, at zero, first crack, 30 kN and yield loads. An impact hammer is used to induce vibration in the slab at each stage and the response is recorded using the piezo-accelerometers attached to the top surface of the slab and the strain gauges under the tensile reinforcement bars at the mid-span section (Figure 6). The signals are processed using HHT. The data from the accelerometers and the strain gauges show similar features. The results show that the frequency decreases as the degree of damage increases with a total reduction of 23% in the fundamental frequency from the healthy stage to the yield stage, which is significant. It is also observed that the major reduction in the frequency occurs between the first crack and 37.5% of the yield load where the tensile cracks tend to propagate rapidly in the thickness direction of the slab, causing significant reduction in stiffness.

### 4.2. Plates

**Figure 7:**
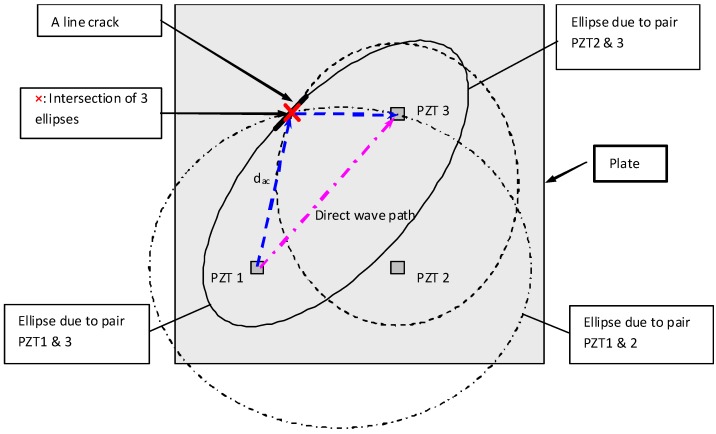
Elliptical loci of possible crack positions (adopted from Figure 1 in [20]).

**Figure 8:**
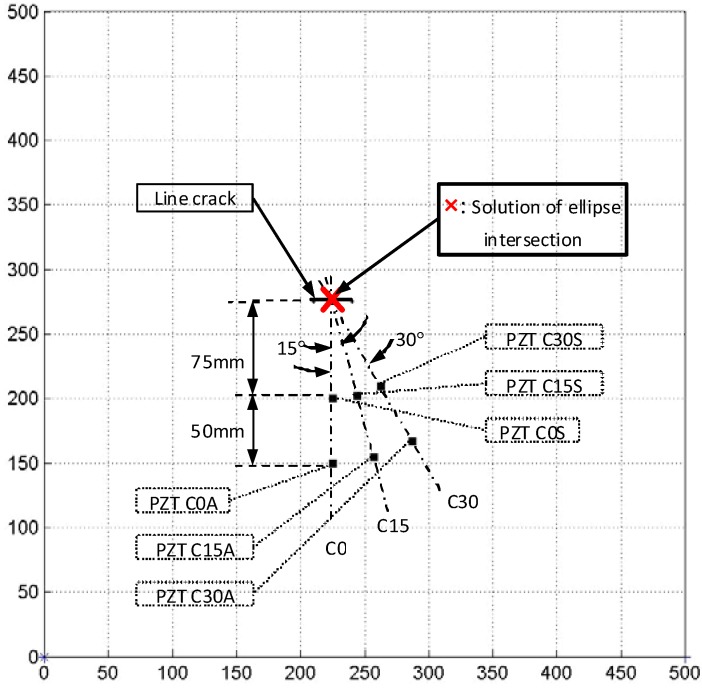
Determination of crack orientation (adopted from Figure 7 in [20]).

**Figure 9:**
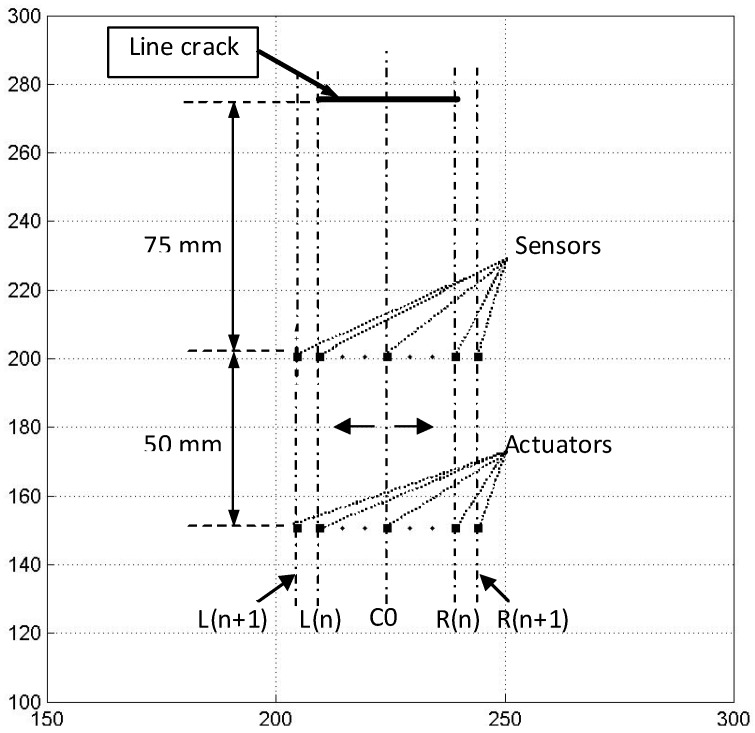
Quantification of damage extent (adopted from Figure 9 in [20]).

In order to detect the defects in a plate structure, the location, orientation and damage extent of a crack need to be identified. Tua *et al.* [20] proposed an elliptical loci method to locate the possible defect positions. Consider an ellipse with PZT1 and PZT3 as the foci, the sum of the distances from any crack on the ellipse to those two foci is constant and equal to the major diameter of the ellipse. In order to identify the exact location of the crack from the infinite solution provided by one ellipse, signals from actuator/sensor pairs at different positions need to be used. This will allow more ellipses to be constructed and their intersection will then provide an estimated location of the crack. A minimum of three ellipses provides an unambiguous estimate of the location, as illustrated in Figure 7. Once the position of the crack is determined based on the intersection of 3 ellipses, the next step is to determine the orientation and extent of the crack. To determine the orientation, two PZTs are placed collinear with the identified crack location at two different positions, for example as shown in Figure 8, C0S is the sensor location and C0A is the actuator location along the line C0. Based on Huygen’s principle and Snell’s Law (incident and reflected angle of the wave are equal), the normal direction of the crack can be determined by monitoring the energy of the crack reflection peak in the spectra for lines of different angles. Once the orientation of the crack is determined, the actuators and sensors may then be shifted in parallel to positions on the left and right of line C0 (see Figure 9). For each position, Lamb wave is then actuated and the spectrum of the signal from the sensor is plotted. The position at which the energy peak first vanishes from the spectrum due to the reflection from the crack indicates the end of the crack. The accuracy of the damage extent can be obtained by using smaller step shifts near both ends. The proposed comprehensive methodology to detect the location, orientation and damage extent of a crack was successfully applied in (1) detection of micro-width cracks of size 330 and 220 μm on an aluminum plate; (2) detecting and locating cracks in-filled with impurities such as grease, araldite epoxy, metallic epoxy and spray paint; (3) detection of a notch in weld.

Compared with plain piezoelectric actuators, IDT has the advantage to excite one single wave mode more strongly than others and hence the excitation can be strong and focused. The optimum design of IDT for the detection of crack in plates was conducted by Jin *et al.* [22]. The design parameters of IDT including finger spacing, finger number, and finger width and length were optimized based on analytical analysis. Using the optimized IDT actuator, two stages were proposed to detect the location and geometry of a crack in a plate, including initial scanning and further refining. Experiments were carried out on 2 mm thickness aluminum plates. The first plate is about 450 × 450 × 2 mm, with a crack line at the center of the plate, parallel to one of the plate boundaries. The crack is around 1.8 mm deep, 1.5 mm wide and 54 mm long. The second plate is about 500 × 500 × 2 mm with two connected crack lines close to the center of the plate at arbitrary angles. The cracks are around 0.7 mm deep, 1 mm wide, and 20 mm and 30 mm long respectively. The results show that by using the designed IDT, the location and the geometry of cracks were efficiently and accurately detected.

As abovementioned, a comprehensive strategy to locate and trace the geometry of cracks in plates has been developed earlier using either plain piezoelectric sensors and actuators [20,23] or IDT [22] based on time of flight analysis of incident and reflected waves. Quek *et al.* [26] compared the two techniques based on results from laboratory experiments. Cracks of different geometries, namely, linear crack, curved crack, and multiple cracks, on aluminum plates were considered. Comparisons of their detection capabilities are also made on a crack of fine width (220 mm) and cracks filled with impurities such as grease, araldite epoxy, metallic epoxy, and spray paint. Both techniques are applied in the case of weld where a notch is induced in the weld on an aluminum plate, and the results obtained for the weld with and without the notch are compared. The results for all cases considered show that both the plain piezoelectric sensors and actuators and IDT techniques can successfully detect and locate the cracks. Plain piezoelectric sensors and actuators are able to provide accurate detection for linear, nonlinear, and piecewise linear cracks, and notch in weld. The geometries are traced with reasonable accuracy. However, IDT has been shown to be more accurate, procedurally simpler, and is an efficient alternative to plain piezoelectric sensors and actuators.

### 4.3. Pipes

**Figure 10:**
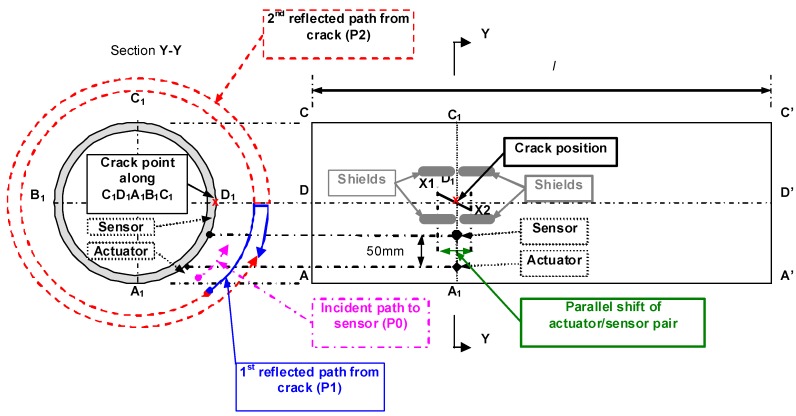
Schematic view of experimental set-up for directed wave propagation in aluminum plate via IDT (adopted from Figure 14 in [23]).

The proposed methodology [20] to detect the location, orientation and damage extent of a crack in a plate was further extended to detect and locate cracks in homogenous pipes by Tua *et al.* [23,24]. By observing the attenuation in the strength of the direct wave incidence at the sensor, the presence of a crack can be determined. As shown in Figure 10, at least four actuation positions with two on each end of the pipe segment of interest are needed to exhaustively interrogate for the presence of cracks. It has been shown experimentally that the detection using a circular piezoelectric actuator and sensor, with dimensions of 5 mm diameter and 0.5 mm thickness, was possible for an aluminum pipe segment of up to at least 4 m in length. The proposed methodology was also explored for an aluminum pipe under more practical situations, such as burying it in sand with only the actuator and sensor positions exposed. Experimental results obtained showed the feasibility of detecting the ‘concealed’ crack on pipes buried in sand.

## 5. Repair of Structures with Smart Materials

Wang *et al.* [50,51,52,53,54,55,56,57,58] developed models to study the use of piezoelectric materials in structural repair, including mechanical models for the repair of cracked or delaminated beam or plate structures under a variety of load conditions. The placement and size of the piezoelectric layer to provide an optimal voltage design with respect to minimizing/removing the stress singularity at the tip of cracks and delaminations were also investigated.

### 5.1. Beams

**Figure 11:**
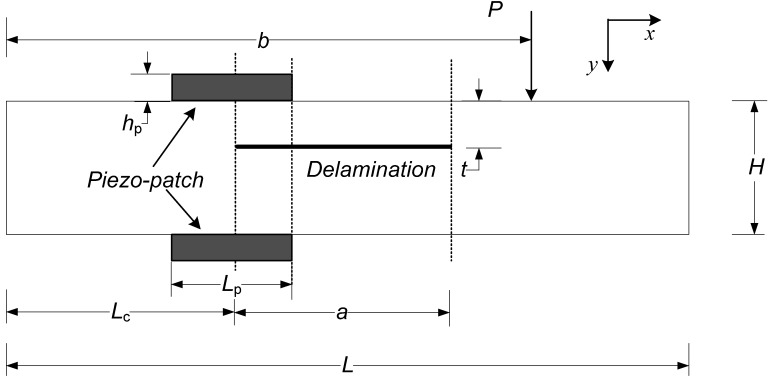
Repair of delaminated beam via piezoelectric patches (adopted from Figure 1 in [56]).

Wang and Quek [53] presented a simple model as shown in Figure 11 for the repair of delaminated beams subjected to concentrated static load via piezoelectric patches. The delaminated beam is subjected to a static load *P* and is to be repaired using piezoelectric patches. The material and geometric parameters of the delaminated beam are denoted as *E* for the Young’s modulus of the host beam, *T* its width, *H* its thickness, *t* the thickness of the upper delaminated layer and *a* the length of the delamination. Their analysis revealed that sliding mode fracture was induced at the tips of the delamination under flexural action of the beam, leading to stress singularity. A simple repair methodology via piezoelectric patches was introduced to remove this singularity. The concept is to balance the axial force induced in the delaminated beam during bending by applying a counteracting force via piezoelectric patches. To repair the beam, two piezoelectric patches with the same thickness $h_{p}$ and length $L_{p}$ are attached. Similar studies were also conducted for the repair of notched beam under static load [51] and dynamic load [52], notched column under axially compressive static load [54], and delaminated beams under compressive force [55], based on simple Euler-Bernoulli beam theory.

**Figure 12:**
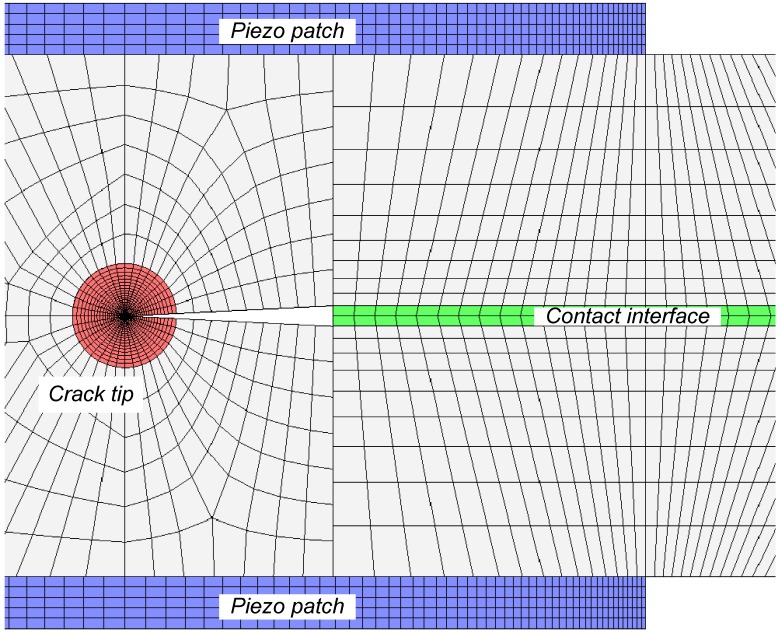
FE discretization for crack tip, piezoelectric patches and contact interface (adopted from Figure 3 in [56]).

**Figure 13:**
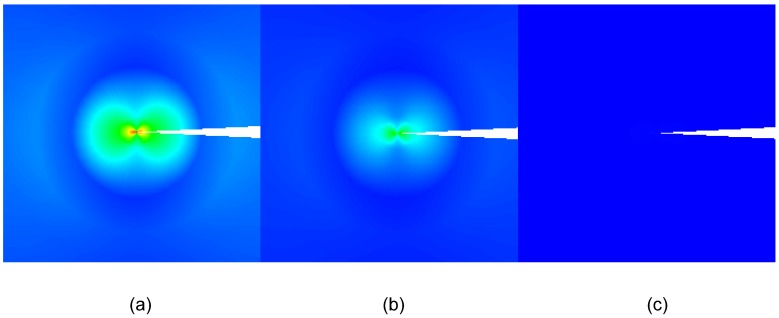
Von-Mises stress field around crack tip with different applied voltages on piezoelectric patches (a) 0 V, (b) 300 V and (c) 480 V (adopted from Figure 5 in [56]).

Duan *et al.*[56] conducted a FE study to verify the repair methodology proposed by Wang and Quek [53]. In FE modeling, the interface of the two layers separated by the delamination is modeled as two surfaces without penetration. The mesh used for the model is shown in Figure 12. Special attention is paid to the FE discretization of crack tip, piezoelectric patches and contact interface. To model the singularity of the crack tip [122], the “sweep meshing” technique is used, which can generate the focused elements having a quad-dominated shape. The number of finite elements with circular area in (*r*, *θ*) directions is 36 × 24. To simulate the coupled thermal and displacement behavior, 20-node quadrilateral, plane strain elements are adopted for the piezoelectric patch and the beam. The repair of the delaminated beam as shown in Figure 11 with concentrated force applied at *b* = 50 mm is studied. With the increase in applied voltage on the piezoelectric patches, the crack is virtually removed as can be confirmed by the von-Mises stress field around the crack (Figure 13). At 0 V, the stress reaches as high as 216 MPa at the tip of the crack as shown in Figure 13 (a). However, as the voltage is increased, the stress decreases to 100 MPa at 300 V as shown in Figure 13 (b) and 150 Pa at 480 V as shown in Figure 13 (c). The repair effect of piezoelectric patches is clearly illustrated numerically.

In order to repair the delaminated beam subject to dynamic loading, Wu and Wang [57] proposed a close-loop feedback control repair methodology using piezoelectric patches. The electromechanical characteristic of the piezoelectric material is employed to induce a local shear force above the delamination area via an external voltage, which is designed as a feedback of the deflection of the vibrating beam to remove the stress singularity around the delamination tips. Moreover, the FE method is employed to verify the effectiveness of the proposed design and repair methodology for delaminated beams with various sizes and alignments of delaminations. The FE results show that although larger repair coefficients could reduce the vibration deflection of the delaminated beam, the stress singularity cannot be erased simultaneously.

### 5.2. Plates

**Figure 14:**
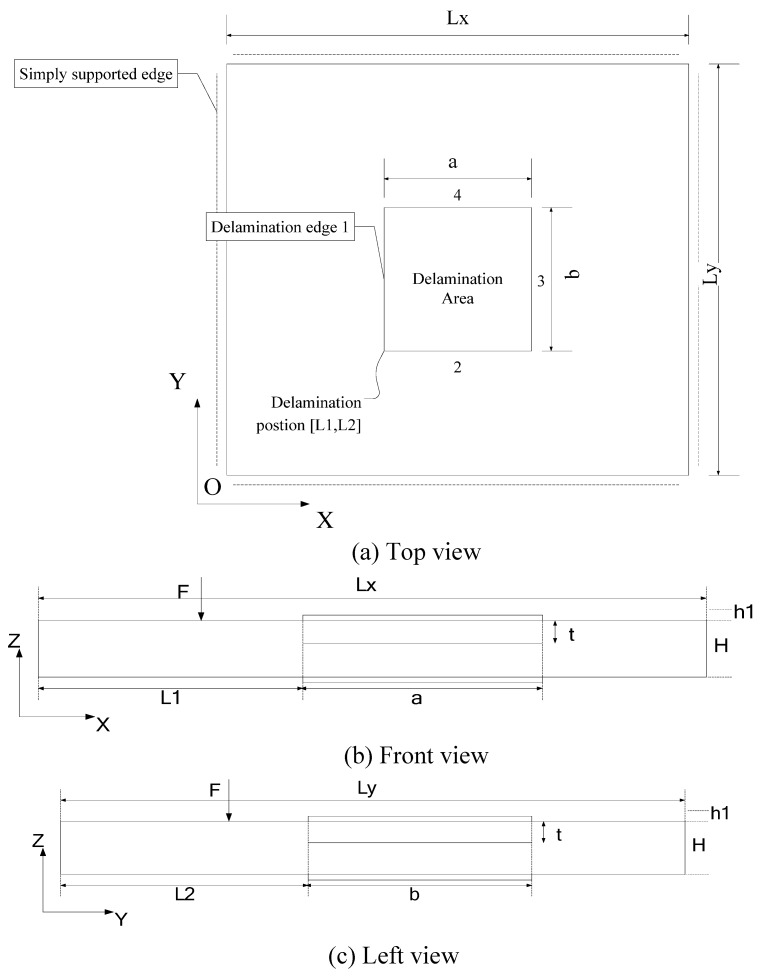
A delaminated plate structure (adopted from Figure 1 in [58]).

**Figure 15:**
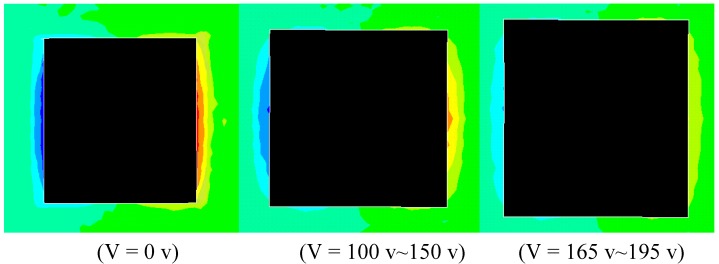
FEM simulation of shear stress distributions along delamination edges with different voltages (adopted from Figure 4 in [58]).

Wu and Wang [58] proposed a repair methodology for a delaminated plate under a static loading with piezoelectric patches. The methodology is developed through an analytical model and the FE method. A delaminated square plate subjected to a static load is shown in Figure 14. A single rectangular delamination is only considered in this study, with the delamination edge numbering illustrated in Figure 14 (a). Figure 14 (b) and Figure 14 (c) illustrate the cross sections of the delaminated area within the XZ and YZ planes, respectively. Piezoelectric patches are surface bonded on the delaminated area. A vertical static load F is applied to the surface of the delaminated plate. While a vertical load is applied to the host plate, deflection will take place along the delaminated area. Axial elongation and compression along X- and Y- directions of the two delaminated layers will be induced accordingly due to the bending of the host structure [52]. Because of the tensile and compressive forces induced, shear stress singularity would be initiated at crack joints of the upper and lower layers of the delamination leading to a second fracture mode according to fracture mechanics. Given the induced stress concentration on the delamination edges, piezoelectric patches are thus employed to produce shear forces between the piezoelectric patches and the host delaminated plate by applying voltages to decrease the magnitude of tensile and compressive forces on the upper and lower delamination layers and hence the shear stress singularity could be erased accordingly. The effectiveness of the analytical model in predicting tensile/compressive force distribution along the delamination layer and voltages applied to the discrete electrodes distributed along Y- direction for reducing the stress concentration along edges 1 and 3 is verified by FE method for a symmetric delaminated plate (L1 = L2 = 0.1 m, a = b = 0.1 m, t = 0.005 m). Figure 15 illustrates the shear stress reduction on edges 1 and 3 when different adjusted voltages are applied to the discrete electrodes. The shear stresses are decreased obviously. From numerical simulations, it is found that larger voltages applied to the discrete electrodes are desirable for shorter delamination. In addition, the voltages will increase when the delamination is close to the mid-face of the host plate.

## 6. Conclusions

Owing to their exceptional mechanical and electric coupling properties, piezoelectric materials hold many potential applications in the fields of structural health monitoring and repair. It is found from research studies that piezoelectric materials have remarkable sensing and exciting capacities. It can be concluded that plain piezoelectric sensors and actuators and IDT have the capability to detect the cracks in beam, plate and pipe with reasonable accuracy. In addition, as an alternative to conventional materials for structural repair, the piezoelectric materials possess promising features (e.g., light weight) to overcome the problems caused by conventional repair methods. The delamination and notch crack in beam and plate structures can be successfully repaired via removing the singularity at the crack tip. Breakthroughs are expected to get rid of the obstacles existing in coupling coefficients and cost for the application in industry scale.
